# Paradoxical embolism caused by totally implantable venous access port: a case report and literature review

**DOI:** 10.3389/fcvm.2025.1581930

**Published:** 2025-08-18

**Authors:** Zonghong Han, Zhongming He, Wenhua Chen, Qi Wang

**Affiliations:** Department of Interventional Radiology, First People’s Hospital of Changzhou, Changzhou, China

**Keywords:** totally implantable venous access port, atrial fibrillation, patent foramen ovale, paradoxical embolism, thrombosis

## Abstract

Totally implantable venous access ports (TIVAPs) are commonly used for patients undergoing chemotherapy and long-term repeated infusions. The incidence of thrombosis is low and rarely leads to serious complications. We report a case of right atrial thrombosis and paradoxical embolism in a 58-year-old male with atrial fibrillation (AF) and patent foramen ovale (PFO) 28 months after TIVAP implantation. The patient presented with dizziness and left limb weakness, subsequent diagnostic imaging revealed right temporal lobe infarction and a mass in the right atrium, who eventually recovered and was discharged after cardiac surgery and anticoagulation. This case highlights the rare but severe complication of right atrial thrombosis associated with TIVAP, particularly in patients with AF and PFO. Proper placement and timely removal of totally implantable venous access ports are crucial to minimize complications. Further research is needed to determine the necessity of anti-coagulation and PFO screening in patients with AF receiving central venous catheters.

## Introduction

Totally implantable venous access ports (TIVAPs) are widely used for patients needing long-term chemotherapy and frequent intravenous infusions. Thrombosis associated with TIVAP is generally manageable with anti-coagulations and rarely leads to severe complications (1). This report presents a unique case of right atrial thrombosis and paradoxical embolism in a patient with a history of atrial fibrillation (AF) and patent foramen ovale (PFO) following TIVAP implantation.

## Case presentation

**Figure 1:**
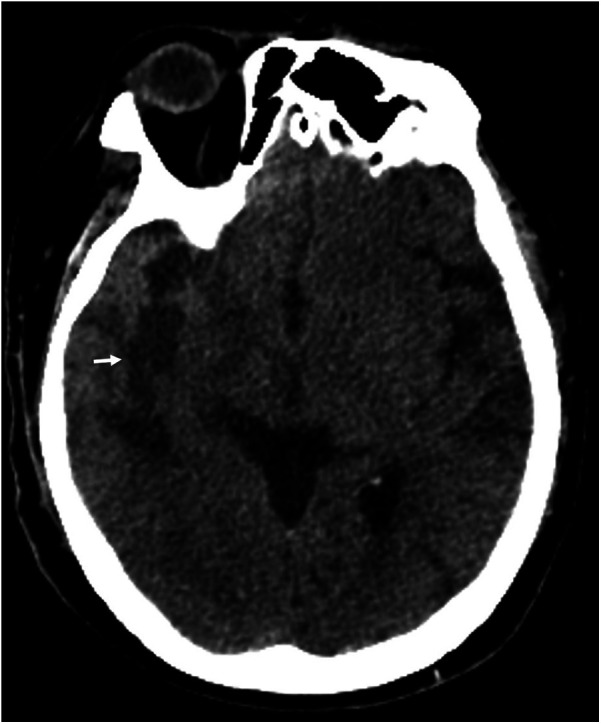
Cranial CT showed right temporal lobe infarction (white arrow).

**Figure 2:**
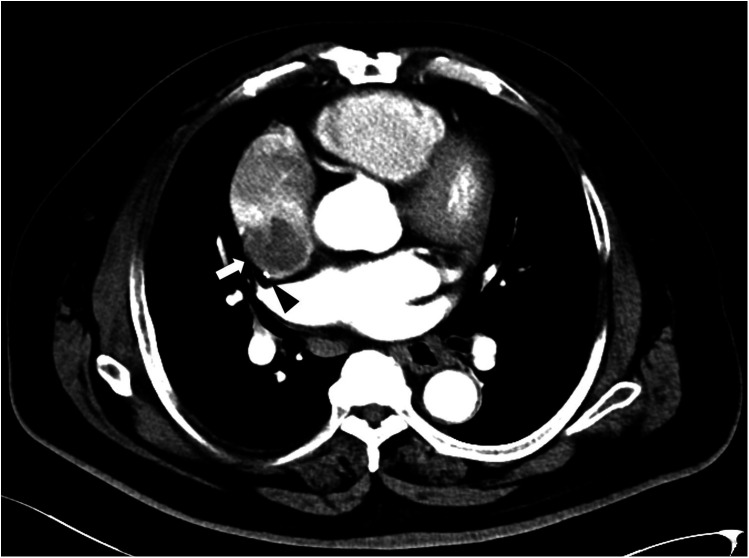
Cardiac CT showed a mass connected to the lateral-posterior wall of the right atrium (white arrow), and the catheter was located behind the mass (black triangle).

**Figure 3:**
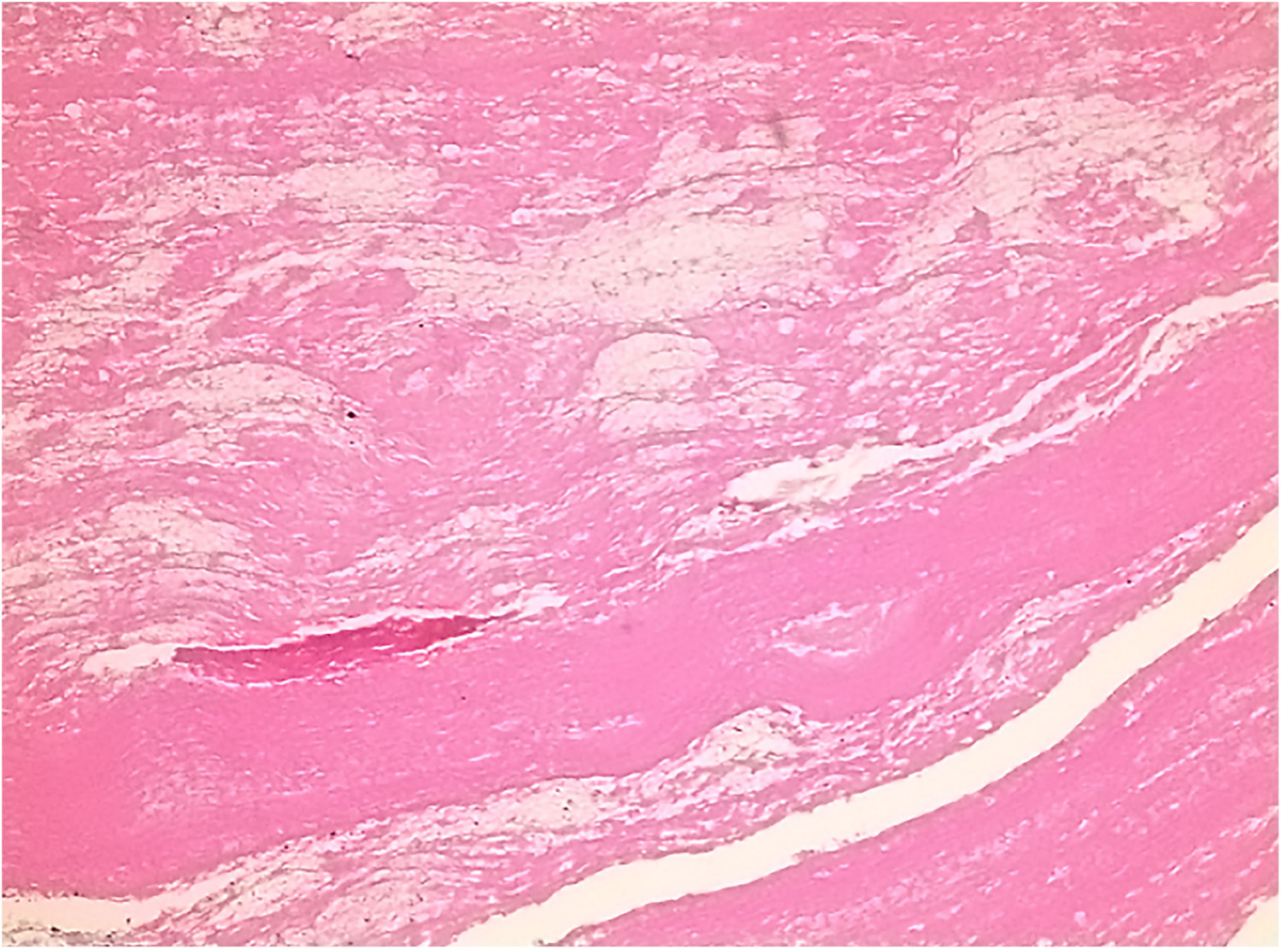
Postoperative pathology showed eosinophilic amorphous material and scattered infiltration of inflammatory cells.

A 58-year-old male with a medical history of hypertension, diabetes and gastro-oesophageal junction low-differentiated invasive adenocarcinoma. Preoperative transthoracic echocardiography revealed a mild degree of heart chambers with a 2 mm PFO and valvular function was normal. Electrocardiogram demonstrated AF and ventricular premature beats. On October 26, 2021, a TIVAP was implanted in the patient's right chest wall for chemotherapy, with the tip positioned at the junction of the superior vena cava (SVC) and the right atrium under fluoroscopy. The patient received six cycles of systemic chemotherapy, comprising intravenous oxaliplatin 100 mg/m^2^ on day 1, oral S-1 80 mg twice daily for 14 days every three weeks. Although the patient has completed the last intravenous chemotherapy in July 2022, the TIVAP was not removed for concern about recurrence. Regular TIVAP maintenance was performed through noncoring needle with 0.9% sodium chloride every four weeks, and the tumor showed complete remission during follow-up. On February 11, 2024, the patient presented to the emergency department with dizziness and left limb weakness lasting two days. Physical examination revealed left nasolabial fold flattening, rightward deviation of the jaw upon mouth opening, and inaccuracy in the left finger nose test. Cranial CT indicated a right temporal lobe infarction (Figure 1). Symptoms improved following the treatment of clopidogrel 75 mg and atorvastatin 20 mg daily. Ultrasound examination showed no abnormalities in the carotid and vertebral arteries, and no thrombosis in the deep and superficial veins of the lower limbs. Transesophageal echocardiography revealed a 44 mm × 23 mm mass in the right atrium and a 2 mm PFO. Further cardiac CT confirmed the mass at the entrance of the SVC into the right atrium, with normal left heart and pulmonary arteries (Figure 2). Differential diagnosis included atrial myxoma, thrombosis, metastatic tumors and infective endocarditis-related vegetations. Two weeks after anticoagulation with vitamin K antagonists, the mass remained static. The TIVAP was used for administering therapy for normal function during this stage. On March 21, 2024, the patient underwent right heart mass resection, PFO repair, TIVAP removal and left atrial appendectomy for reducing the risk of thrombosis in the future. Postoperative pathology showed fibrous tissue without proliferative cells, revealed that the mass was thrombosis (Figure 3). The patient recovered well and was discharged, with no embolic events during follow-up on continuous rivaroxaban 20 mg daily.

## Discussion

Most TIVAP-related thrombi are asymptomatic and often resolve with anticoagulation without causing serious complications, even when symptomatic (1). However, severe conditions, such as right atrial thrombosis and secondary pulmonary embolism, or even paradoxical embolism can occur.

The incidence of catheter-related right atrial thrombosis (CRAT) reported in studies varies from 2% to 29% (2–4). The true incidence may be underestimated as the diagnosis is often not considered in asymptomatic and symptomatic patients, or may be missed by transthoracic echocardiography (4). When atrial thrombus was suspected, transesophageal echocardiography should be performed. Most cases reported in the literature involve patients undergoing hemodialysis, with rare cases associated with TIVAPs (5). Hemodialysis catheters, due to larger diameter and placement in the right atrium to ensure optimal blood flow, are more likely to cause atrial thrombosis through mechanical stimulation and high blood flow (6). The increased risk of venous thrombosis in chronic kidney disease is also associated with underlying hemostatic derangements (7). In contrast, TIVAP catheters are thinner, and the recommended position of the tip is in the lower third of the SVC or at the cavoatrial junction, which may contribute to the lower incidence of right atrial thrombosis. However, the TIVAP tip position is not static and can move as respiratory variation, arm movement and changes in body position (8). When the tip moves, it may contact the right atrial wall and damage the endothelial lining, predisposing the patient to thrombosis formation (2, 9). In this case, the catheter tip was located at the cavoatrial junction, and the thrombosis was connected to the right atrium by a pedicle, likely due to damage caused by the tip's movement. Thrombi in the atrium can enter the systemic or pulmonary circulation, leading to embolic events and corresponding clinical symptoms. AF is a significant cause of atrial thrombosis, usually occurring in the left atrium. The placement of a central venous catheter may theoretically increase the incidence of thrombosis in patients with AF. To our knowledge, the relationship between central venous catheter-related thrombosis and AF has not been studied. This patient had a history of AF but not received anticoagulation for asymptomatic and worried about bleeding despite CHADS-VASc score of 2. Herein, the concomitant AF likely exacerbated thrombosis formation following local damage to the right atrium caused by catheter. It should be noted that the role of AF in catheter-related right atrial thrombus formation remains unclear. Most evidence associates AF with left atrial thrombosis, and the mechanisms linking AF to right atrial thrombosis in the presence of central venous catheters have yet to be elucidated. More clinical cases and studies are needed to confirm this potential relationship.

Research indicates that the incidence of PFO in the adult population ranges from 15% to 35%, with most individuals being asymptomatic (10). PFOs can open under certain physiological or pathological conditions, such as the Valsalva maneuver, coughing, vomiting and right-sided strain (11). Paradoxical embolism, caused by peripheral venous thrombi passing through a PFO, is not uncommon in the literature and poses a significant medical burden to patients (10–12). Studies have shown a higher prevalence of PFO in patients with cryptogenic stroke than in the general population (13). In this case, the paradoxical embolism was likely caused by fragments of the right atrial thrombosis entering the systemic circulation through the PFO, as no other embolic source could be identified. Research indicates significant benefits of percutaneous PFO closure compared to medical therapy alone, especially with careful patient selection.

CRAT can be complicated by sepsis, arrhythmias, pulmonary embolism, or systemic embolism in the case of PFO, and is associated with an overall mortality rate of up to 45%. Current treatment options for CRAT include catheter removal and/or replacement with anticoagulation, thrombolysis, thrombectomy, and catheter-directed interventions. Stavroulopoulos et al. proposed a management algorithm emphasizing catheter removal and recommending anticoagulation as first-line treatment. Moreover, they advised CVC removal and anticoagulation for thrombi smaller than 6 cm, with surgical thrombectomy recommended for larger thrombi, contraindications to anticoagulation, or endocarditis (3). Yang et al. suggested maintaining hemodialysis by replacing catheters and providing oral anticoagulation or antiplatelet therapies as an effective strategy for treating hemodialysis patients with CRAT (2). Rossi et al. recommended systemic anticoagulation with vitamin K antagonists, targeting an International Normalized Ratio of 2.5–3.0, combined with urokinase as a locking solution at the end of each hemodialysis session, although this approach appears successful in only 60% of cases (14). However, these options are based on individual reports and retrospective case series, as there are no clear evidence-based therapeutic guidelines for these thrombi. Given the patient's history of AF, PFO, cerebral infarction and ineffective anticoagulation, surgical treatment was performed after multidisciplinary consultations, and the patient was eventually discharged.

Although this case may be incidental, it highlights the potential risks associated with AF and PFO in patients with TIVAP. When considering CRAT combined with PFO and paradoxical embolism, anticoagulation as first-line treatment. Otherwise, thrombus removal and PFO repair should be considered. Prospective studies could provide valuable insights into optimal strategies for preventing and treating this condition. While TIVAPs provide essential long-term venous access for patients undergoing chemotherapy, they are not without risks. Proper placement, regular maintenance, and timely removal of these devices are crucial to minimize complications.

## Data Availability

The original contributions presented in the study are included in the article/Supplementary Material, further inquiries can be directed to the corresponding author.

## References

[1] Tabatabaie O Kasumova GG Eskander MF Critchlow JF Tawa NE Tseng JF. Totally implantable venous access devices: a review of complications and management strategies. Am J Clin Oncol. (2017) 40:94–105. 10.1097/coc.0000000000000361 28106685

[2] Yang H Chen F Jiao H Luo H Yu Y Hong HG Management of tunneled-cuffed catheter-related right atrial thrombosis in hemodialysis patients. J Vasc Surg. (2018) 68:1491–8. 10.1016/j.jvs.2018.02.039 29804743

[3] Stavroulopoulos A Aresti V Zounis C. Right atrial thrombi complicating haemodialysis catheters. A meta-analysis of reported cases and a proposal of a management algorithm. Nephrol Dial Transplant. (2012) 27:2936–44. 10.1093/ndt/gfr739 22187317

[4] Clark JR Hoffman SC. Incidence of catheter-associated right atrial thrombus detected by transthoracic echocardiogram. Echocardiography. (2021) 38:435–9. 10.1111/echo.14987 33523518

[5] Zhang Y Shi J Li JJ Zhang L Li Y. Systemic thrombolysis and anticoagulation therapy for catheter-related right atrial thrombosis caused by TIVAP: a case report and review of the literature. J Vasc Access. (2022) 23:313–7. 10.1177/1129729821989159 33506722

[6] Lok CE Huber TS Lee T Shenoy S Yevzlin AS Abreo K KDOQI Clinical practice guideline for vascular access: 2019 update. Am J Kidney Dis. (2020) 75:S1–s164. 10.1053/j.ajkd.2019.12.001 32778223

[7] Bernardo J Oliveira J Gameiro J Outerelo C. Asymptomatic pulmonary thromboembolism due to hemodialisys catheter thrombosis: case series and literature review. CEN Case Rep. (2023) 12:318–22. 10.1007/s13730-022-00757-4 36574198 PMC10393924

[8] Nickel B Gorski L Kleidon T Kyes A DeVries M Keogh S Infusion therapy standards of practice, 9th edition. J Infus Nurs. (2024) 47:S1–s285. 10.1097/nan.0000000000000532 38211609

[9] Chick JF Reddy SN Bhatt RD Shin BJ Kirkpatrick JN Trerotola SO. Significance of echocardiographically detected central venous catheter tip-associated thrombi. J Vasc Interv Radiol. (2016) 27:1872–7. 10.1016/j.jvir.2016.07.013 27659895

[10] Teshome MK Najib K Nwagbara CC Akinseye OA Ibebuogu UN. Patent foramen Ovale: a comprehensive review. Curr Probl Cardiol. (2020) 45:100392. 10.1016/j.cpcardiol.2018.08.004 30327131

[11] Li JD Xu N Zhao Q Li B Li L. Multiple paradoxical embolisms caused by central venous catheter thrombus passing through a patent foramen ovale: a case report. World J Clin Cases. (2024) 12:842–6. 10.12998/wjcc.v12.i4.842 38322689 PMC10841136

[12] Petrea RE Koyfman F Pikula A Romero JR Viereck J Babikian VL Acute stroke, catheter related venous thrombosis, and paradoxical cerebral embolism: report of two cases. J Neuroimaging. (2013) 23:111–4. 10.1111/j.1552-6569.2010.00568.x 21281383

[13] Mojadidi MK Zaman MO Elgendy IY Mahmoud AN Patel NK Agarwal N Cryptogenic stroke and patent foramen Ovale. J Am Coll Cardiol. (2018) 71:1035–43. 10.1016/j.jacc.2017.12.059 29495983

[14] Rossi L Covella B Libutti P Teutonico A Casucci F Lomonte C. How to manage catheter-related right atrial thrombosis: our conservative approach. J Vasc Access. (2021) 22:480–4. 10.1177/1129729820922703 32410490

